# cAMP protein kinase phosphorylates the Mos1 transposase and regulates its activity: evidences from mass spectrometry and biochemical analyses

**DOI:** 10.1093/nar/gkt874

**Published:** 2013-09-28

**Authors:** Nicolas Bouchet, Jérôme Jaillet, Guillaume Gabant, Benjamin Brillet, Luis Briseño-Roa, Martine Cadene, Corinne Augé-Gouillou

**Affiliations:** ^1^Innovation Moléculaire Thérapeutique, EA 6306, UFR Sciences Pharmaceutiques, Parc Grandmont, Université François Rabelais, 37200 Tours, France, ^2^Centre de Biophysique Moléculaire, UPR 4301 CNRS, Rue Charles Sadron, 45071 Orléans, France, ^3^Laboratoire Universitaire de Biodiversité et Ecologie Microbienne, IUT de Quimper, Université de Bretagne Occidentale, 6 rue de l’Université, 29000 Quimper, France and ^4^Biologie Cellulaire de la Synapse, INSERM U789, Ecole Normale Supérieure, 46 rue d'Ulm, 75005 Paris, France

## Abstract

Genomic plasticity mediated by transposable elements can have a dramatic impact on genome integrity. To minimize its genotoxic effects, it is tightly regulated either by intrinsic mechanisms (linked to the element itself) or by host-mediated mechanisms. Using mass spectrometry, we show here for the first time that MOS1, the transposase driving the mobility of the *mariner Mos1* element, is phosphorylated. We also show that the transposition activity of MOS1 is downregulated by protein kinase AMP cyclic-dependent phosphorylation at S170, which renders the transposase unable to promote *Mos1* transposition. One step in the transposition cycle, the assembly of the paired-end complex, is specifically inhibited. At the cellular level, we provide evidence that phosphorylation at S170 prevents the active transport of the transposase into the nucleus. Our data suggest that protein kinase AMP cyclic-dependent phosphorylation may play a double role in the early stages of genome invasion by *mariner* elements.

## INTRODUCTION

The transposition of genetic elements is potentially genotoxic for the host cell despite being an essential tool for perpetuating genome plasticity, and thus for its evolutionary potential. This means that tight regulation of transposition is essential to maintain the balance between maintaining active transposons in host genomes and preventing the damage they could cause by potentially lethal DNA rearrangements. Studies of the relationships between transposable elements and the host genome have revealed diverse examples of the regulations that can be achieved through intrinsic, emergent or host-mediated mechanisms. Intrinsic regulation can be due to topological constraints (1), to the poisoning of transposition reactions by the overproduction of transposase (2,3) or to strong negative complementation between active transposases and the product of mutated alleles (2). Transposon silencing, in which transposition is restricted by various epigenetic pathways (4,5), is probably the best known of the regulatory mechanisms that have recently emerged. Little is known about host-mediated regulatory mechanisms, which are triggered when a transposon invades a naive genome that may be nonspecialized mechanisms widely used by eukaryotic cells. They could therefore consist of posttranslational modifications (PTMs), which are often found to drive the regulation of protein activity. Such modifications could alter the cellular localization of a protein, trapping the modified protein in a cellular compartment that is inappropriate for its activity. Alternatively, PTM could modify the stability of the protein, shortening its residence time in the cell, and thereby limiting its activity. Finally, PTM could directly alter protein activity, making it unable to promote any biochemical process.

Over 200 types of PTMs have been identified in eukaryotes so far. The most common ones include (i) phosphorylation, a key reversible modification used as a regulatory mechanism in virtually every process in eukaryotic cells (6), and (ii) acetylation/methylation, which is particularly involved in the regulation of chromatin expression through histone modification (7). While up to 30% of all proteins may be phosphorylated, so far posttranslational phosphorylation has only been explored for the transposase of the *P*-element of Drosophila (8). The possible regulation of the *mariner Mos1* transposase by phosphorylation was recently suggested by *in silico* prediction and alanine mutagenesis scanning (9), but has not been formally demonstrated.

To address this issue, we applied mass spectrometry (MS) methods to a MOS1 protein produced by insect cells, in a cell context free of transposition events. We did this to detect basal regulatory pathways, which could account for the inbuilt regulation of MOS1 when *Mos1* enters a naive genome. We found that MOS1 was phosphorylated at two residues: S2 and S170. The kinase responsible for S2 phosphorylation has not yet been identified, whereas S170 is strongly phosphorylated by the protein kinase AMP cyclic-dependent (PKA). Using biochemical approaches, we investigated the role of S2 and S170 phosphorylation (pS170) in MOS1 activity. The S2 phosphorylation has little or no effect. In contrast, the pS170 produces a dramatic decrease in MOS1 activity, which becomes unable to promote the transposition of a pseudo-*Mos1* element *in vitro*. We identified the mechanism by which pS170 prevents transposition, and showed that it impairs the assembly of the paired-end complex (PEC), probably by steric hindrance and/or charge repulsion. In addition, we found that the cellular distribution pattern of pS170 MOS1 was different from that of the wild-type protein.

## MATERIALS AND METHODS

### MBP-MOS1 production and purification

MBP-MOS1 was produced using the pVL1392 baculovirus transfer vector and the BaculoGoldTM baculovirus expression system (BD Biosciences), and then extracted from baculovirus-infected *Sf21* cells. The fusion protein was purified onto a maltose binding resin (New England Biolabs, NEB) as described previously (10). After purification, only the full-length transposase was obtained (Pflieger *et al*., submitted for publication). His-MBP-MOS1 was similarly produced and purified from crude extract by affinity chromatography on a HiTrap column (GE Healthcare), and then on an amylose resin (NEB). The purified His-MBP-MOS1 was the kind gift of Guillaume Carpentier. For Xa-factor digestion, 5 µg of His-MBP-MOS1 or of MBP-MOS1 were incubated for 2 h at 25°C in 15 µl with 2 mM CaCl_2_, 5 mg of bovine serum albumin (BSA; NEB) and 1 µg of Xa-factor protease (NEB). The samples were boiled and loaded onto discontinuous 4–10% sodium dodecyl sulphate-polyacrylamide gel electrophoresis (SDS-PAGE), and then stained with ProQ Diamond (Invitrogen) or Coomassie blue. The Pro-Q Diamond phosphoprotein gel stain provides a method for selectively staining phosphoproteins in polyacrylamide gels, according to the manufacturer’s recommendations.

MBP-MOS1, MBP-S170A and MBP-S170D were produced and purified from *Escherichia coli* as described previously (10). MBP-S2D was obtained from the pMal-MOS1 by site-directed mutagenesis (the oligonucleotides sequence is provided in Supplementary Table S1). MBP-S2D was produced and purified, as was MBP-MOS1.

### MS analyses

#### Determination of the phosphorylation stoechiometry by MS

*Sf21*-expressed MBP-MOS1 (10 µg) was incubated at 30°C for 45 min, with or without 15 U/µl lambda phosphatase (Sigma), in the recommended buffer consisting of 50 mM Tris/HCl, pH 7.5, 2 mM MnCl_2_, 5 mM dithiothreitol (DTT) and 0.1 mM Na_2_EDTA. The samples were acidified with formic acid at a final concentration of 1% (v/v) before desalting with a C4 ZipTip from Millipore (Billerica, MA, USA). Desalted samples were analyzed by direct infusion in a 4-GHz MaXis ultra-high-resolution Q-TOF mass spectrometer from Bruker Daltonics (Germany) equipped with an electrospray ion source. Acquisitions were carried out in positive ion MS mode over a 300–3000 *m/z* range, with a nebulizer gas pressure of 0.3 bars. The drying gas flow and temperature were 4 L/min and 180°C, respectively. The acquisition rate was 1 Hz corresponding to spectra summations of 5494. External calibration was performed with ESI-L Low Concentration Tuning Mix (Agilent Technologies). ElectroSpray Ionization - High Resolution Mass Spectrometry (ESI-HRMS) spectra were processed and charge-deconvoluted using DataAnalysis 3.1 software (Bruker Daltonics) and the MaxEnt algorithm.

#### Phosphorylation site identification by Liquid Chromatography Mass Spectrometry (LC-MS)

Cysteine reduction/carbamidomethylation was performed on MBP-MOS1 by a 30-min treatment at 56°C with a final concentration of 1 mM Tris(2-carboxyethyl)phosphine hydrochloride (TCEP) in 50 mM ammonium bicarbonate buffer, followed by 30 min alkylation at room temperature in the dark with 5 mM iodoacetamide. Alkylated MBP-MOS1 was cleaved for 4 h at 37°C with porcine trypsin at a 1:20 w:w trypsin:protein ratio. The tryptic phosphopeptides were enriched from the cleavage products by IMAC using iron-coated PHOS-Select™ metal chelate beads from Sigma in accordance with the manufacturer's instructions. Bound peptides were eluted using 1% phosphoric acid and analyzed by nanoLC-nanoESI-IT-MS/MS. Experiments were performed on the UltiMate™ 3000 NanoRSLC System (Dionex, Sunnyvale, CA, USA) connected to a Bruker HCT Ultra PTM Discovery ion trap equipped with an online nanoelectrospray ion source. The LC-MS setup was controlled by the Bruker HyStar™ software version 3.2. Phosphopeptides were preconcentrated online on a Dionex Acclaim Pepmap100, 100 µm × 2 cm, C18 5 µm 100 Å reverse-phase precolumn and separated on a nanoscale Acclaim Pepmap100, 75 µm × 15 cm, C18 2 µm 100 Å column at a flow rate of 200 nl/min using a 5–45% gradient of acetonitrile in 0.1% formic acid. Mass spectra were acquired in positive ion mode from *m/z* 100 to 2000 for MS, and from *m/z* 100 to 3000 for MS/MS. The MS acquisitions were carried out at enhanced resolution (8100 *m/z* units per second). Collision-induced dissociation (CIP) and electron-transfer dissociation (EDT) fragmentations were performed in separate nanoLC-IT-MS/MS runs.

### Phosphorylation of bacterially expressed MBP-MOS1 transposase

Seventy-five micrograms of purified MBP-MOS1 (from *E. coli*) were incubated with 170 units of cAMP-dependent protein kinase A catalytic subunit (PKA, Promega) and either γ^32^P ATP (25 µCi) or ATP (3.5 mM) in 40 mM Tris HCl, pH 7.4, and 20 mM Mg acetate, for 15 min at 30°C. Samples were then desalted on a protein desalting spin column (PIERCE). MBP5 (NEB) phosphorylation was achieved in a similar fashion. Radiolabeled proteins were analyzed on a 4–10% discontinuous SDS-PAGE. The gel was fixed, dried and exposed for 48 h on a Phosphor Screen (STORM Molecular Dynamics). For experiments to compare phosphorylated and nonphosphorylated MBP-MOS1 (from *E. coli*), the nonphosphorylated protein was subjected to the same procedure in the absence of PKA. They were designated +PKA and −PKA, respectively. Similar treatments were used to obtain phosphorylated MBP-S170A.

### *In vitro* transposition assays

*In vitro* transposition reactions were performed using pBC-3T3 as both the donor DNA and the target, as previously described (11). Basic transposition reaction mixtures contained 10 mM Tris-HCl (pH 9), 50 mM NaCl, 20 mM MgCl_2_, 0.5 mM EDTA, 100 ng of BSA, 80 nM MBP-MOS1 (±PKA as specified), and 600 ng of supercoiled pBC-3T3 in a volume of 20 µl. The reactions were allowed to proceed for 30 min at 30°C, before 10 µl of stop solution (0.4% SDS, 0.4 µg/µl proteinase K) was added. The reaction mixtures were then incubated at 37°C for a further 30 min, and then at 65°C for 10 min. The products were phenol-chloroform extracted, and ethanol precipitated with 1 µg of yeast tRNA using standard techniques. Ten percent of the reaction mixture was co-transformed in 45 µl of electro-competent JM109 *E. coli*. Bacteria were grown at 37°C for 1 h in super optimal Broth (SOC) media. Appropriate dilutions of each reaction mixture were plated on LB-tetracycline (12.5 µg/ml) agar and Luria Broth (LB)-chloramphenicol (80 µg/ml) agar to score for transposition frequency. The transposition frequency was the number of Tet^R^ colonies divided by the number of Chloram^R^ colonies + the number of Tet^R^ colonies.

### Excision assays

Basic excision reaction mixtures contained 10 mM Tris-HCl (pH 9), 50 mM NaCl, 20 mM MgCl_2_, 0.5 mM EDTA, 0.5 mM DTT, 100 ng of BSA, 600 ng of supercoiled pBC-3T3 and 80 nM MBP-MOS1 from *E. coli* (±PKA as specified), in a volume of 20 µl. The reactions were allowed to proceed at 30°C for various periods (as indicated), before adding 2 µl of stop solution (loading buffer). The reaction mixtures were then incubated at 65°C for 10 min. Each reaction was analyzed by overnight electrophoresis at 2.7 V/cm on a Tris-Acetate-EDTA (TAE)-buffered 1% agarose gel. After electrophoresis, the gel was stained with ethidium bromide (0.3 mg/ml), and photographed on a transilluminator.

### Electrophoretic mobility shift assays

Binding reactions were carried out in 50 mM NaCl, 0.5 mM DTT, 10 mM Tris (pH 9), 5% glycerol, 1 µg of sonicated salmon sperm DNA and 100 ng of BSA. Each 20-µl reaction mixture contained ^32^P-labeled double-stranded oligonucleotide with the *Mos1* 3′inverted terminal repeat (ITR) sequence, and purified MBP-MOS1 from *E. coli* (±PKA as specified). To detect single-end complexes (SEC2), 0.2 pmol of uncleaved 3′ITR (UC-ITR) with 10 pmol of purified MBP-MOS1 were used [corresponding to ITR70 in (12)], and reactions were carried out in 5 mM EDTA for 30 min at 4°C. To detect PECs, 2.5 pmol of precleaved 3′ITR (PC-ITR) with 2.5 pmol of purified MBP-MOS1 were used (3), and reactions were carried out in 5 mM MgCl_2_ for 30 min at 30°C. The reaction products were separated using 6% native polyacrylamide/0.25× Tris-Borate-EDTA (TBE) gels (30:0.93 acrylamide-bisacrylamide) containing 5% glycerol. Gels were run at 200 V for 3 h, and then exposed for 16 h on a Phosphor Screen (STORM Molecular Dynamics). The sequences of the PC- and UC-ITRs are given as Supplementary data.

### Cellular localization of green fluorescent protein-fusion proteins

#### DNA constructs

Schneider-2 (S2) cells were transfected with the pCS2 expression vector containing either the green fluorescent protein (GFP) alone or the GFP in C-terminal fusion with the wild-type MOS1, or the S170A and the S170D mutants. The pCS2-GFP and pCS2-MOS1-GFP plasmids were a kind gift from Marie-Véronique Demattei (13). pCS2-S170A-GFP and pCS2-S170D-GFP were obtained from the pCS2-MOS1-GFP by site-directed mutagenesis (the oligonucleotide sequence is provided in Supplementary Table S1). pCS2, in which the fusion proteins are expressed under the control of a pCMV (mammalian) promoter, was used to prevent the overproduction of the fusion proteins, which could have altered the MOS1-GFP signal (13).

#### Cell manipulation

S2 cells were handled according to the Supplier's instructions (Invitrogene) and maintained at 26°C in Schneider-2 media with 10% (v/v) heat-inactivated fetal calf serul (FCS) (GIBCO) and 1.0 mg/ml PSG (Penicillin-Streptomycin-Glutamine, GIBCO). Transfections were done using calcium phosphate. Briefly, 1 µg of DNA was mixed with 1× HBS buffer (21 mM hydroxyethyl piperazineethanesulfonic acid (HEPES), pH 7.1, 137 mM NaCl, 5.5 mM Dextrose, 50 mM KCl, 0.7 mM Na_2_HPO_4_). The 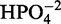/DNA mix was then mixed with CaCl_2_, and incubated at room temperature (RT) for 30 min. The 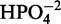/DNA/Ca^2+^ suspension was added to 0.5 × 10^6^ cells seeded in concanavalin-A–coated plates, and incubated for 24 h. Cells were washed, resuspended in media containing 1% FCS/700 µM CuSO_4_ and incubated for 72 h. The medium was removed from the plates, which were then washed with phosphate buffered saline (PBS).

#### Imaging

Cells were fixed in PBS/2% paraformaldehyde at RT for 15 min, and then permeabilized with PBS/1% (w/v) Triton-X100 for 10 min. Hoechst 33342 dye was added at 1/250 and the cells then incubated for 30 min. Cells were washed with PBS and mounted for imaging. Single focal plates (1 μ) were acquired in a Leica DM5000B microscope, a CSU10 (Yokogawa) spinning disk head, a CoolSNAP HQ2 (Photometrics) CDD camera and the software MetaMorph (v7.6.1.0, Molecular Devices). No postacquiring imaging processing was done except for adjusting the overall contrast of the images.

## RESULTS

The DNA transposition of *mariner* elements is now a well-established process, which involves several steps: (i) sequence-specific binding of a transposase dimer to the ITR present at one transposon end (forming a SEC2); (ii) pairing of the transposon ITRs to form a PEC; (iii) cleavage of both DNA strands at each transposon end; and (iv) integration of the transposon at a new locus. The *Mos1* transposase (MOS1) is a 345-AA protein containing two domains: an N-terminal domain, which promotes the assembly of MOS1 dimers and their subsequent binding to *Mos1* ITR (10), and a C-terminal domain, which promotes catalytic functions (DNA strand cleavages and transfers) (14). It should be noted that *mariner* transposition is believed not to require specific cellular host factors, as it occurs *in vitro* (11) as well as in a variety of organisms, such as *E. coli* (15), *Caenorhabditis elegans* (16), *Bombyx mori* (17) and *Drosophila melanogaster* (18). Intriguingly, *Mos1* still displays a restricted host range, suggesting that MOS1 may retain some inbuilt regulatory elements.

### MOS1 expressed in insect cells is phosphorylated

**Figure 1.**
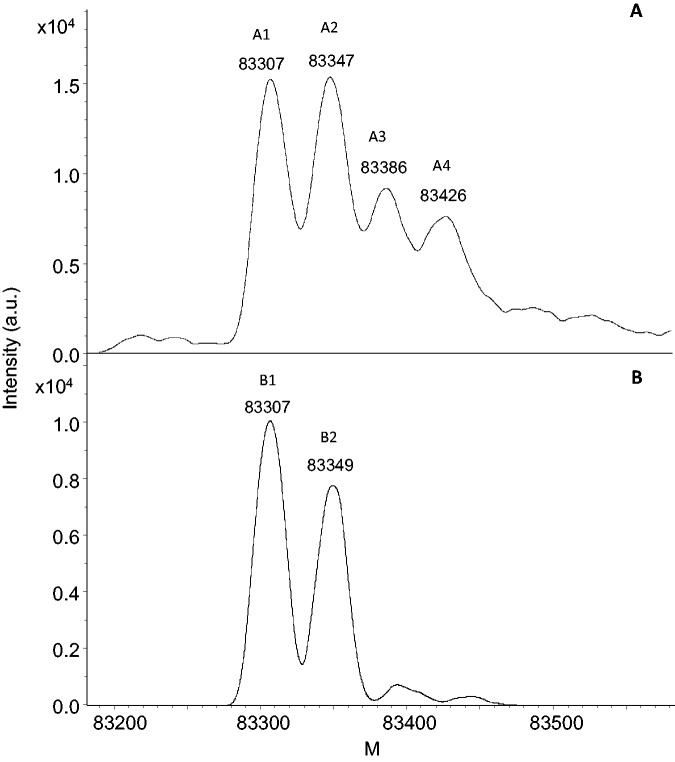
Deconvoluted high-resolution mass spectra of MBP-MOS1. Ten micrograms of *sf21*-expressed MBP-MOS1 was incubated without (**A**) or with (**B**) lambda phosphatase in the buffer recommended by the manufacturer (Sigma). Untreated samples gave four peaks. A1 and A2 correspond to nonphosphorylated MBP-MOS1; treated samples displayed only two peaks, B1 and B2, corresponding to A1 and A2, respectively. A3 and A4 are the phosphorylated versions of A1 and A2, respectively. The molecular mass after deconvolution is given in Da for each protein peak. a.u.: arbitrary units.

We expressed MOS1 using a baculovirus/insect cell (*sf21*, cells derived from the Fall Armyworm *Spodoptera frugiperda*) system, as this involves the expression of MOS1 in a cellular context similar to that of the native host (*Drosophila* species). Moreover, this expression system is efficient for phosphorylation and displays a high level of protein production. MOS1 was N-terminally fused with maltose binding protein (MBP). The MBP-tag was used both to achieve high-quality purification and to enhance MOS1 stability (15). MS was used to analyze MOS1 PTMs (predicted mass without the N-terminal methionine: 83 258.23 Da). Purified MBP-MOS1 from infected *Sf21* extracts was incubated with or without lambda (λ) phosphatase. Samples were analyzed by direct infusion ESI in an ultra-high-resolution Q-TOF mass spectrometer. Spectra from the untreated sample reveal four main peaks with masses of 83 307 (A1), 83 347 (A2), 83 386 (A3) and 83 426 Da (A4), respectively (Figure 1A). Following treatment with λ phosphatase, we obtained spectra showing two peaks with masses of 83 307 Da (B1) and 83 349 Da (B2). The mass difference between A3 and A1 (79 Da) and between A4 and A2 (79 Da), together with the disappearance of A3 and A4 in response to λ phosphatase treatment, is consistent with an apparent phosphorylation stoichiometry value of one. This indicates that A3 and A4 are monophosphorylated forms of MBP-MOS1, which can therefore be defined as a phosphoprotein (Figure 1B). The relative abundance of the two phosphorylated forms (A3 and A4) to the unphosphorylated ones (A1 and A2, corresponding to B1 and B2, respectively) suggests that the amount of MBP-MOS1 phosphorylation is limited.

As the peak of an acetylated protein is shifted by 42 Da by covalently bound acetyl group by comparison with the unmodified protein, the mass differences between A1 and A2 and between B1 and B2 (40–42 Da) could therefore be due to acetylation. The mass profile suggests that about half of the MBP-MOS1 analyzed was produced as an acetylated protein. Furthermore, a mass difference of +49 Da was measured between the theoretical mass of MBP-MOS1 (83 258 Da without N-terminal methionine) and the observed mass (A1 = 83 307 Da). This mass difference could correspond to a point mutation (N to Y or H to W) or to extensive oxidation of methionine and/or tryptophan residues.

**Figure 2.**
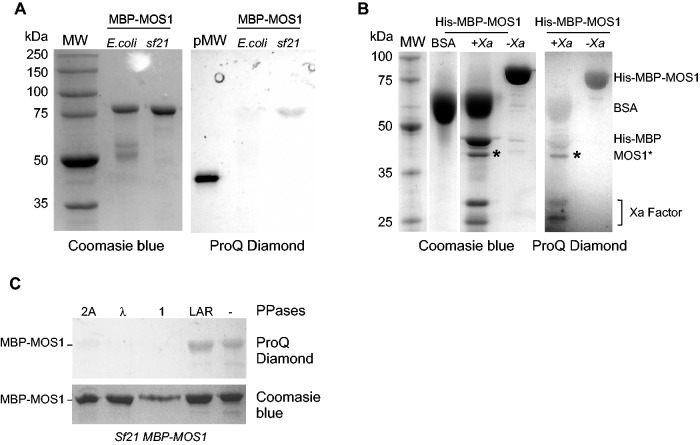
*Sf21*-expressed MBP-MOS1 is phosphorylated. (**A**) Comparison of purified MBP-MOS1 expressed in bacteria (*E. coli*) and in insect cells (*sf21*). Molecular weights (MW) are given in the left margin (in kDa). The staining method is indicated below the gels: Coomassie blue staining (left) and ProQ Diamond staining (right). MW: molecular weight standards. pMW: the phosphorylated marker corresponds to the phosphorylated ovalbumin (≈45 KDa) with five phosphorylated residues/protein, from Peppermint Stick (Invitrogen). (**B**) Purified His-MBP-MOS1 (expressed in *sf21*) is analyzed after cleavage by the Factor Xa protease (+Xa) or not (−Xa). BSA is added to the reaction mix to obtain efficient Factor Xa cleavage, according to the manufacturer’s instructions (NEB). Molecular weights (MW) are given in the left margin (in kDa). The staining method is indicated below the gels. An asterisk pinpoints MOS1 after Factor Xa cleavage. The corresponding proteins are indicated in the right margin. (**C**) Purified MBP-MOS1 (expressed in *sf21*) is analyzed after treatment by various phosphatases (PPases), as indicated at the top of the figure. The staining method is indicated in the right margin: ProQ Diamond staining (top) and Coomassie blue staining (bottom).

As a link between pseudo-phosphorylation and the alteration of transposition activity has been proposed for MOS1 (9), we focused our attention on the phosphorylation of the transposase. First, equal amounts of MBP-MOS1 purified from *Sf21* cells or *E. coli* were analyzed by SDS-PAGE, and stained with ProQ Diamond (Invitrogen) and Coomassie blue. The ProQ Diamond dye is used to detect phosphorylated proteins. As expected, ProQ Diamond staining revealed a signal for MBP-MOS1 produced from *Sf21* cells, but not from bacteria (Figure 2A). We then checked whether the phosphorylation observed was carried by the MOS1 or MBP moiety of the fusion protein; to do this we took advantage of the presence of a cleavage site for the Factor Xa protease between the MBP and the MOS1 sequences of the fusion protein. To increase the resolution between MBP and MOS1 protein gel bands, the His-MBP-MOS1 fusion produced in *sf21* cells was used rather than the MBP-MOS1. Following cleavage of the fusion product by factor Xa protease, two proteins of ∼44 (His-MBP) and 41 kDa (MOS1) were observed. Coomassie blue staining revealed that MOS1 was present as a less intense band than His-MBP, whereas ProQ Diamond labeled the MOS1 band much more intensively than His-MBP (Figure 2B). These findings indicate that MOS1 was phosphorylated, whereas His-MBP was not. The intensity of the signal obtained with ProQ Diamond staining in these experiments was consistent with the MS data, and confirmed that the level of MOS1 phosphorylation was low. Finally, we used various protein phosphatases to find out whether the phosphorylated residues consisted of serine, threonine or tyrosine. MBP-MOS1 produced in *sf21* cells was treated with phosphatases 1 or 2A (serine and threonine dephosphorylation), λ phosphatase (serine, threonine and tyrosine dephosphorylation) or LAR phosphatase (for tyrosine dephosphorylation). Our data indicated that only serines and threonines could be involved in MOS1 phosphorylation (Figure 2C), since the LAR phosphatase had no effect on MBP-MOS1 phosphorylation status.

### MOS1 phosphorylated residue(s)

**Figure 3.**
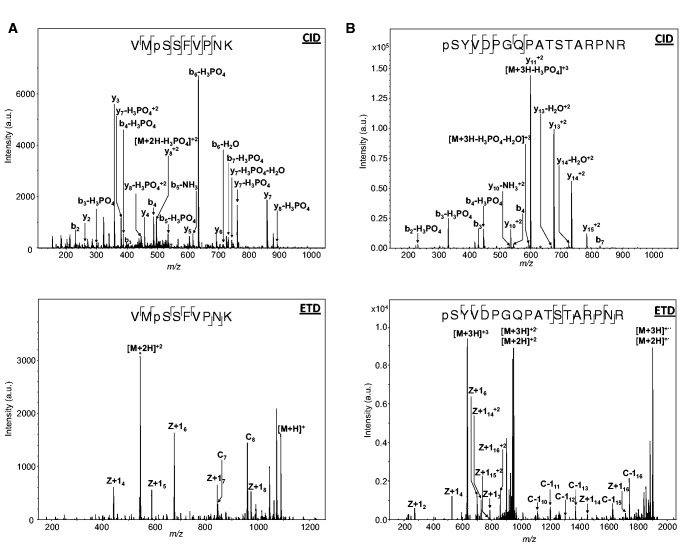
Evidence for pS2 and pS170 in MOS1. (**A**) CID and ETD fragmentation mass spectra of the phosphorylated MBP-MOS1 [VMSSFVPNK] peptide. The precursor ion is a doubly charged peptide at m/z 544.7. (**B**) CID and ETD fragmentation mass spectra of the phosphorylated MBP-MOS1 [SYVDPGQPATSTARPNR] peptide. The precursor ion is a triply charged peptide at m/z 639.9.

Phosphorylation sites identification was done by MS. Cysteine reduction/carbamidomethylation was performed on baculovirally expressed MBP-MOS1, followed by alkylation in the dark. Alkylated MBP-MOS1 was cleaved with porcine trypsin. The tryptic phosphopeptides were enriched from the cleavage products by IMAC. After elution, peptides were analyzed by nanoLC-IT-MS/MS CID and ETD. Spectra show that two serines are phosphorylated in MOS1 (Figure 3): S2 and S170 (numbering with the N-terminal methionine). The localization of phosphorylation sites on S2 and S170 was unambiguously determined by a combination of CID and ETD experiments. A search for acetylation on the N-terminus and on lysine side chains of the protein did not find any acetylation site. However, no signal was observed for the N-terminal peptide. Of the 12 methionines present in the sequence, seven are detected as partially oxidized, which could explain the mass difference of +49 Da between the expected and observed masses of the MBP-MOS1 protein. No mutation was identified, despite a sequence coverage of 85.7%.

**Figure 4.**
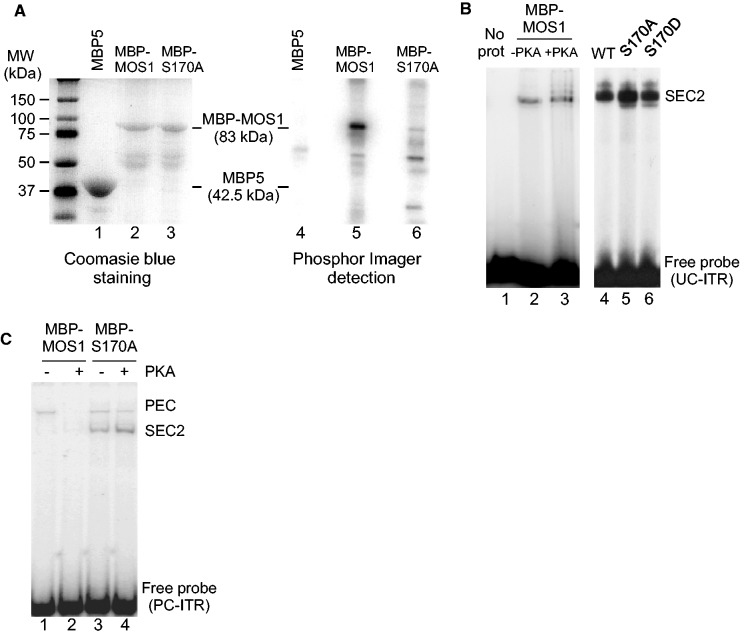
MBP-MOS1 phosphorylation by PKA regulates PEC assembly. (**A**) Equal amounts of MBP-MOS1 (lanes 2 and 5) and MBP-S170A (lanes 3 and 6) were loaded, together with MBP5 (NEB) (lanes 1 and 4). MBP5, MBP-MOS1 and MBP-S170A, expressed in bacteria, were used as substrates for PKA, in the presence of γ^32^P ATP (lanes 4–6). The proteins of interest (and their calculated molecular weights) are indicated in the middle. Molecular weight markers (MW) are given in the left margin (kDa). The manufacturer describes MBP5 as a 42.5-kDa protein. Under labeling conditions (lane 4), no signal was observed at the corresponding MW, indicating that the PKA is unable to phosphorylate the MBP5. However, a faint signal was detected at 65 kDa, suggesting the presence of a contaminant protein in the MBP5 sample. (**B**) EMSAs were performed using the MBP-MOS1 incubated with PKA (lane 3) or not (lane 2) and with the *Mos1* 3′ITR as a probe, under conditions allowing SEC2 assembly to occur. The probe alone was loaded in lane 1. EMSAs performed using untreated proteins are shown in the right panel: MBP-MOS1 (lane 4), MBP-S170A (lane 5) and MBP-S170D (lane 6). The complexes formed between MBP-Transposases and the ITR are indicated in the right margin. (**C**) EMSAs were performed using MBP-MOS1 treated (lane 2) or not (lane 1) by PKA and with the *Mos1* 3′ITR as a probe, under conditions allowing paired-ends complex (PEC) assembly. EMSAs were performed using MBP-S170A treated (lane 4) or not (lane 3) by PKA, under identical conditions. The complexes formed between MBP-Transposases and the ITR are indicated in the right margin.

While S170 has previously been predicted to be a phosphorylated residue (9), that is not the case for S2. Kinases capable of phosphorylating S2 and S170 were investigated using the GPS (Group-based Phosphorylation Scoring Method) software (19) (http://csbl.bmb.uga.edu/∼ffzhou/gps_web/predict.php). No kinase was predicted as capable of phosphorylating S2, whereas S170 could be phosphorylated by at least 10 different kinases. Among these, PKA had the best prediction score, and the sequence surrounding S170 (165-PKRKK**S**YV-172) perfectly overlapped the PKA consensus phosphorylation site (RxxT/S). To corroborate these analyses, we performed *in vitro* phosphorylation of MBP-MOS1 produced in bacteria. Bacterially expressed MBP-MOS1 and MBP5 (NEB) were used in equal concentrations as substrates for PKA in the presence of γ^32^P ATP. After electrophoresis, MOS1 was confirmed to be efficiently phosphorylated by PKA treatment, since MBP-MOS1 was strongly labeled during the assay, whereas MBP was not. MBP-S170A (corresponding to an alanine substitution at the S170 residue, making the position 170 no more phosphorylable) was treated in a similar way, and displayed drastically reduced protein labeling by PKA (Figure 4A). These findings demonstrated that MOS1 is phosphorylated at position S170 by PKA.

### The phosphorylation of the S170 residue controls MOS1 activity

The relationship between PKA phosphorylation and MOS1 activity was then investigated. *In vitro* genetic ‘hop’ experiments were done using the pBC-3T3 plasmid, as the transposon donor and the target plasmid, and purified MBP-MOS1 transposase. Transposition events were recovered by bacterial transformation and selection for the appropriate antibiotic resistance marker (tetracycline resistance, as a gain-of-function landmark for transposition) (10). The assays were done using MBP-MOS1 phosphorylated by PKA or not. Two controls were used: MBP-MOS1 treated in the absence of PKA (to evaluate the impact of treatment conditions, i.e. incubation and desalting) and untreated MBP-MOS1. As previously published, untreated MBP-MOS1 promotes pseudo-*Mos1* (3T3) transposition with a rate of 10^−^^4^ (Table 1). We found that MBP-MOS1 incubated in absence of PKA promoted 3T3 transposition with a rate of 10^−^^5^, whereas the PKA phosphorylated MBP-MOS1 was unable to promote transposition. The difference between untreated and incubated transposases (both of them nonphosphorylated) reflected a decrease in specific activity as a result of treatment. The involvement of the S170 residue in MOS1 regulation by PKA phosphorylation was similarly analyzed. *In vitro* transposition assays were performed using the *E. coli* purified MBP-S170A mutant, treated with or without PKA. S170A treated by PKA was still able to promote the 3T3 transposition with a rate of 10^−^^6^, similar to that obtained without PKA. Another confirmation was obtained using the MBP-S170D mutant, a pseudo-phosphorylated MOS1 mutant that is unable to promote the 3T3 transposition. These data confirm a strong relationship between MOS1 transposition regulation by PKA and the S170 residue.

We went on to identify which stage(s) of *Mos1* transposition cycle are controlled by MOS1 phosphorylation, focusing first on single-end and PEC assembly. Electrophoretic mobility shift assays (EMSAs) were performed with MBP-MOS1 (±PKA) and the *Mos1* 3′ITR as a probe, using conditions appropriate for the detection of either SEC2 or PECs, as previously described (3,10). SEC2 assembly was done using UC-ITR, under noncatalytic conditions (EDTA and 4°C). Our data show that MBP-MOS1 phosphorylation did not prevent SEC2 assembly (Figure 4B, lanes 2–3), since we got the same signal regardless of the protein used (MBP-MOS1 ± PKA). Similarly, both S170A and S170D were able to promote SEC2 assembly (Figure 4B, lanes 5−6). We concluded that PKA phosphorylation at S170 was not able to prevent either SEC2 assembly, or the MOS1 dimerization that precedes ITR binding. PEC assembly was assayed using PC-ITR, under catalytic conditions (MgCl_2_ and 30°C) (Figure 4C). No signal corresponding to the PEC was detected with the PKA-treated MBP-MOS1 (lane 2), whereas a weak signal was detected with the MBP-MOS1 (-PKA) (lane 1), showing that MBP-MOS1 phosphorylation interferes with PEC assembly. The role of pS170 in the PEC assembly was confirmed by probing the ability of the S170A mutant to promote PEC assembly. The same signal was obtained regardless of the treatment (±PKA), and the relative amount of SEC2 versus PEC was greater than with the wild-type enzyme (Figure 4C, lanes 3−4). As we have demonstrated previously, UC-ITRs only supported SEC2 assembly. In contrast, PC-ITRs can support SEC2 or PEC assembly: the ‘choice’ between these two complexes relies on the amount of *Mos1* transposase relative to ITR. For equal or less amounts of transposase, the PEC was detected, whereas in excess of transposase the SEC2 was detected (3). In Figure 4C, conditions were chosen to detect the PEC. The pathway of PEC assembly involves several steps (20): first, the MOS1 dimer interacts with a single ITR to make a SEC2, then the dimer is reorganized to form a complex competent to recruit the second ITR and form the PEC. The results observed in lane 2 (traces of SEC2 as in lane 1, and no PEC) suggested that the SEC2 did not ‘accumulate’ when MOS1 was phosphorylated at position S170 but was lost during the dimer reorganization that appeared abortive. In contrast, no difference was observed between S170A treated or not by PKA (compared lanes 3 and 4), and the overall behavior of the protein was different from that of the wild type. S170A was mainly blocked in the initial dimer conformation, promoting SEC2 assembly instead of PEC. Little dimer reorganization was allowed, accounting for the SEC2/PEC ratio detected. This was consistent with the fact that the promotion of *Mos1* transposition by S170A is less efficient than that produced by the wild-type protein. The inability of S170D to promote PEC assembly (Supplementary Figure S1) is consistent with the data reported above. Hence, our data support the conclusion that the phosphorylation of the MOS1 S170 residue by PKA controls MOS1 activity by preventing PEC assembly.

**Figure 5.**
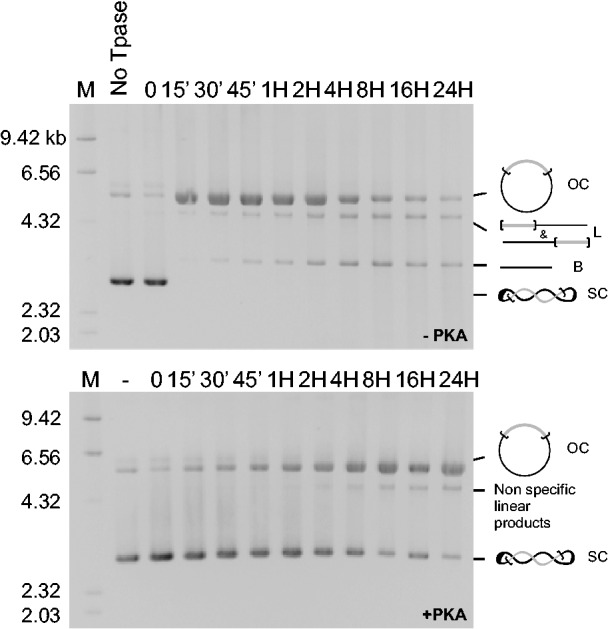
MBP-MOS1 phosphorylation by PKA prevents a pseudo-*Mos1* excision. Time course analyses of excision were performed using MBP-MOS1 treated (bottom panel) or not (top panel) by PKA, and the pBC-3T3 plasmid as the donor of transposon. The assays were performed at 30°C, and the resulting products were loaded onto agarose gel. Molecular weight markers are indicated on the left (in kb). The various products are depicted on the right, and their positions on the gel are indicated. First strand nicking at one transposon end generated an open circular product (OC). Second strand nicking linearized the donor (L), yielding the single-end break product. A similar sequence of nicks at the other transposon end yielded the double-end break products, which consist of the plasmid backbone (B) plus the excised transposon fragment. After excision, the transposon could be reintegrated, making it difficult to be detected in electrophoresis. SC: supercoiled donor.

In the *Mos1* transposition cycle, PEC assembly precedes the first and second strand cleavages that promote excision (10). We therefore confirmed our EMSA data using *in vitro* excision assays. Time-course analyses of the excision assays were performed using MBP-MOS1 (±PKA), and the pBC-3T3 as a transposon donor. Our data show that MOS1 phosphorylated by PKA is unable to promote excision, as shown by the continued presence of the supercoiled donor, and the absence of backbone release even after incubating for 24 h (Figure 5). The presence of linear products had previously been shown to be owing to nonspecific cleavages of pBC DNA by *Mos1* transposase (10). Under similar conditions, there was little difference between the activity of the MBP-S170A (±PKA), and this activity was much lower than that of the MBP-MOS1 without PKA (Supplementary Figure S2). Taken together, these findings confirm that the phosphorylation of MOS1 by PKA prevents *Mos1* transposition by inhibiting the assembly of PECs, and consequently further DNA cleavages.

### The S2 residue

As previously indicated, the kinase responsible for S2 phosphorylation is unknown, making it difficult to perform the same studies as that performed for p170. Nevertheless, the use of pseudo-phosphorylated residues (D for pS) was demonstrated to be convenient (see our data with S170D), we prepare the S2D mutant. We assume that this mutant may help to describe the consequences of S2 residue phosphorylation. *In vitro* genetic ‘hop’ experiments were done using either purified MBP-MOS1 transposase or the MBP-S2D mutant and the pBC-3T3 plasmid, as the transposon donor and the target plasmid. Our data show that the S2D mutation has no effect on the transposase activity since the MBP-S2D promotes the pseudo-*Mos1* (3T3) transposition with a rate of 7.10^−^^5^ (for recall, the wild-type transposase promotes the 3T3 transposition with a rate of 10^−^^4^).

### pS170 controls the cellular localization of MOS1

**Figure 6.**
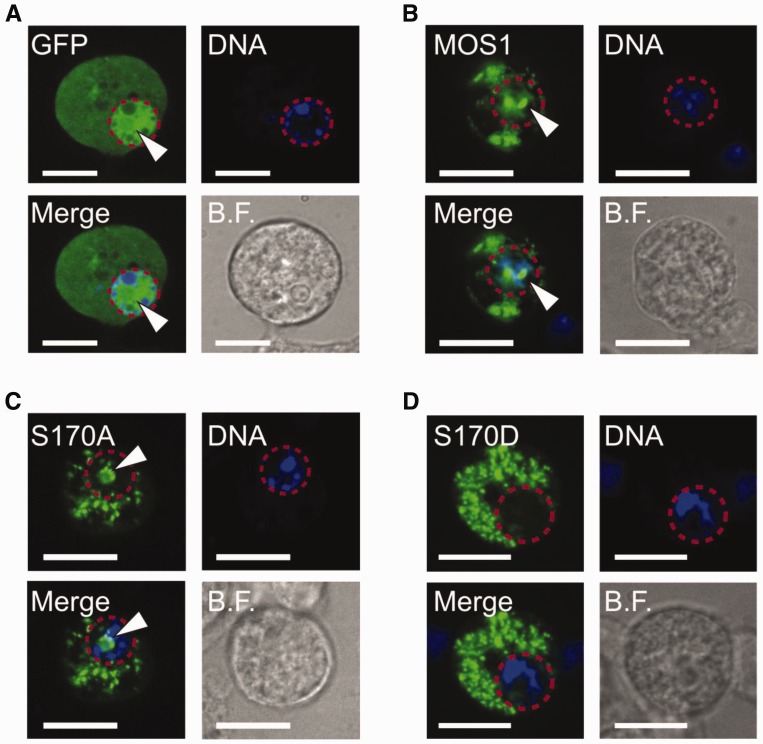
pS170 relocates MOS1 in the cytoplasm. Schneider-2 cells were transfected with pCS2-GFP (**A**), pCS2-MOS1-GFP (**B**), pCS2-S170A-GFP (**C**) or with pCS2-S170D-GFP (**D**). Cells were fixed with 2% paraformaldehyde, stained with Hoechst dye, and examined for fluorescence. Hoechst stained nuclei are blue in color, and surrounded by a red dotted line. GFP fluorescence is shown in green. BF: bright field. Scale bar represents 10 μm. Nuclear localization is indicated by white arrow heads.

In a recent publication, Demattei *et al*. have proposed that the nuclear localization of MOS1 is dependent on the presence of both an SV40 NLS-like motif (position: AA 168 to 174), and a dimerization subdomain located within the first amino terminal helix-turn-helix motif of the protein (13). Because the S170 residue is located in the middle of the SV40 nuclear localization sequence (NLS)-like motif, we wondered whether its phosphorylation could also affect the cellular localization of MOS1, in addition to impair PEC assembly. We used the S170D pseudo-phosphorylated transposase expressed as a protein fusion with GFP. Given that S170D adequately mimics the behavior of phosphorylated MOS1, we assumed that it could be used to illustrate the intracellular trafficking of the phosphorylated protein. The ability of MOS1, S170A and S170D to enter the nucleus of insect cells (embryonic *D.melanogaster*, S2 cells) was investigated by transient expression of plasmids coding for the full-length transposases fused at their C-terminal end with GFP (Figure 6). Transfection with a plasmid expressing the GFP alone was performed as a control. GFP is found in both the cytoplasm and the nucleus of S2 cells. Its size places it below the size exclusion limit of the nuclear pore complex, thus allowing it to diffuse passively into the nucleus (Figure 6A). In contrast, the molecular mass (70 kDa) of the MOS1-GFP, S170A-GFP or S170D-GFP fusion proteins means that they cannot passively diffuse into the nucleus. If these proteins are found within the nucleus, this must, therefore, result from active transport (21). As previously reported (13), some of the intracellular MOS1 are localized in the nucleus of S2 cells (Figure 6B). Similar data were obtained with the nonphosphorylable S170A mutant (Figure 6C), supporting the idea that a serine at position 170 is not a strict requirement for a functional NLS. However, the pseudo-phosphorylated S170D mutant was excluded from the nucleus (Figure 6D), indicating that phosphorylation of MOS1 at residue 170 does prevent its active transport to the nucleus.

## DISCUSSION

Little is known about the regulation of transposases by PTM, despite the fact that this could reveal the intimate relationships that bind DNA transposons and host-genomes. Here, we report on the PTM undergone by a eukaryotic transposase in a transposon-free cell system. This situation presumably corresponds to what happens when a transposon enters a naive genome during either natural horizontal transfer propagation or biotechnical DNA transfer. Using the *mariner Mos1* element as a model, we show that the transposase undergoes two kinds of PTM: acetylation and phosphorylation. MBP-MOS1 is ∼50% acetylated, but the site of acetylation could not be precisely determined. In addition, a small fraction of the transposase produced in insect cells is phosphorylated, and MOS1 phosphorylation never exceeds an apparent stoichiometry of one. While MOS1 acetylation remains to be explored, we decided to focus the present report on MOS1 phosphorylation, a regulatory mechanism involved in virtually every process in eukaryotic cells.

We have demonstrated that PKA phosphorylation at residue S170 (pS170) plays a key role in regulating MOS1 activity, since the pS170 transposase is unable to promote transposition. This effect relies on the inability of the phosphorylated transposase to form the PEC and thus the subsequent transposition steps. On the other hand, pS170 does not impair the ability of MOS1 to form SEC2. By extension, this finding also shows that pS170 does not alter MOS1 dimerization. The crystal structure of the *Mos1* synaptic complex locates the S170 residue of one monomer in a structure called the ‘clamp loop’ (residues 162–189), which locks the PEC by interacting with the linker region (residues 113–125) of the second monomer (22). More specifically, S170 is part of a short β-strand (β4: residues 169–172), which forms an antiparallel β-sheet with β1 of the linker of the other monomer. The position of S170 in the *Mos1* transposase is thus consistent with the effect of its phosphorylation by PKA. pS170 displays more steric hindrance and more negatively charges, which may be sufficient to locally destabilize the clamp loop/linker interface and thereby prevent the assembly of the PEC. In addition, neither MOS1 dimerization nor the SEC2 assembly involved the S170 region, which accounts for the lack of effect of pS170 with regard to these activities. In addition to controlling the transposition of Mos1, phosphorylation at S170 modifies the intracellular route of the transposase, trapping the protein in the cytoplasm. These results are not contradictory, since the S170 residue is located in a part of the MOS1 protein involved in both activities: the nuclear localization and, on transposition, the assembly of the synaptic complex. In a cellular context, the phosphorylation of MOS1 at position 170 could have a double effect: it prevents the translocation to the nucleus of the transposase and then it prevents the excision of *Mos1*-related elements integrated into the genome. The control of the cellular localization by PTM involving phosphorylation is a well-known process, which has been extensively described for transcriptional regulators; however, we report here for the first time that mariner transposases are prone to this kind of regulation.

**Table 1.** Transposition rate of the various transposases used

Transposase	MOS1	MOS1 − PKA	MOS1 + PKA	S170A	S170A − PKA	S170A + PKA	S170D	S2D
Transposition rate	10^−4^	10^−5^	nD	3.10^−5^	10^−6^	10^−6^	nD	7.10^−5^

To characterize the activity of the various b-MOS1 transposases used in this study, we used an *in vitro* genetic ‘hop’ experiment, which is the most sensitive test available as yet (3). Purified transposases were incubated with the pBC-3T3 plasmid, which was used both as the transposon donor and the target plasmid. This plasmid contains the pBR322 tetracycline resistance gene (without promoter) framed by two *Mos1* ITRs. This reconstitutes a pseudo-*Mos1* element named 3T3. We took advantage of the fact that the *cat* gene (present in the pBC backbone) is a hotspot for *Mos1* integration (35). Consequently, transposition events were revealed by promoter tagging, the tetracycline resistance being activated through the *cat* gene promoter. Transposition events were recovered by bacterial transformation with selection for tetracycline resistance, as a gain-of-function landmark for transposition. MOS1, S170A, S170D and S2D: *Mos1* transposases (wild type and mutants) produced by *E. coli*; ±PKA: the corresponding transposases have been incubated with (+) or without (−) PKA before to be used in the transposition assays; nD: nondetectable.

During the past decade, the relationships between eukaryotic DDE enzymes (transposases, integrases and recombinases) activity and kinase activity have been addressed. The rationale underlying these studies is that the DDE recombinases cause DNA double-strand breaks (DSBs), which must be repaired if the cell is to survive. These repairs involve specific kinases [the ataxia telangiectasia mutated (ATM) or DNA-dependent protein kinases (DNA-PK)], and their involvement in regulating recombinases activity has been studied. Beall and colleagues (8) have shown that the activity of the *P*-element transposase may be down-regulated by ATM-kinase phosphorylation. Until recently, this was the only evidence of a phosphorylation being directly involved in regulating a recombinase activity. A novel component of the DSBs repair pathway, SETMAR, has recently been identified. SETMAR is a SET (histone methylase) and a *mariner* transposase fusion protein found only in anthropoid primates (23,24). Recently, the phosphorylation of SETMAR following DNA damage has been demonstrated (25). SETMAR is phosphorylated at a single position (S495) leading to enhancement of its DSB repair activity and repression of its replication fork restart activity. The main kinase involved is Chk1, known for its role in cell cycle control (26). Interestingly, S495 is located in the transposase moiety of SETMAR. Sequence alignments between MOS1 and SETMAR show that the corresponding amino acid in MOS1 is in fact S170. The sequence surrounding S170 in MOS1 (165-PKRKK**S**YV-172) does not overlap a Chk1 consensus phosphorylation site (LxRxxS/T). In contrast, the sequence surrounding S495 in SETMAR (489-YDNRRR**S**A-496) overlaps a PKA consensus phosphorylation site (RxxT/S), suggesting that SETMAR is not only sensitive to Chk1 regulation but could also be controlled by PKA. More generally, a PKA phosphorylation consensus is found around S170 for all the transposases of the *mauritiana* subfamily (to which MOS1 belongs), and also for the *mariner* transposases of the *cecropia* (to which SETMAR belongs) and *mellifera/capitata* subfamilies (Supplementary Figure S3). In contrast, the *mariner* transposases of the *irritans* subfamily (to which HIMAR1 belongs) do not contain a PKA phosphorylation consensus site in the vicinity of S170.

The perfect conservation of the PKA consensus phosphorylation site around S170 in the *mauritiana* subfamily suggests that pS170 regulation plays a crucial role within the subfamily, whatever the host species. In contrast to Chk1 or the ATM and DNA-PK kinases, PKA is a cytoplasmic kinase and is the primary mediator of cAMP action in eukaryotic cells. As a consequence, biological conditions that activate PKA will concomitantly decrease the activity of MOS1 (and of the related transposases), before it can promote transposition and trigger DNA damage. PKA is implicated in diverse pathways that regulate cell survival and apoptosis in response to metabolic and growth signals, and it can elicit opposite effects depending on the cell type and situation. In addition to these activities, PKA has been shown to be involved in the infectious cycle of several viruses (27–29). Several studies indicate that cross talk occurs between the DNA damage checkpoint and PKA activity in yeast. These findings support a model in which DNA damage can regulate the PKA pathway to phosphorylate substrates that act to restrain mitosis (30–32). Too little is known about PKA involvement in DNA damage repair to allow us to suggest that PKA has a role in controlling *Mos1* transposition in cells where the transposon is established. In contrast, we can say that PKA phosphorylation probably plays a role in the early stages of genome invasion by *mariner* elements. *Mariner* transposases that are PKA-sensitive might be downregulated before the element expands (regardless of the DSBs they trigger), thus preventing any major amplification of the element. This might be the case for *Mos1* elements that are present in low copy numbers in their host-genome (up to 30) (33) and of related elements of the *mauritiana* subfamily. In contrast, *mariner* transposases that are not PKA sensitive may not be downregulated at this stage, potentially leading to major amplification of the element, before any induced regulatory mechanisms can take place. This could have happened in the case of *Himar1* elements (of which there are thousands of full copies in the *Haematobia irritans* genome) (34). Hence, our data suggest new clues about the genome invasion dynamics of *mariner* elements. The transposition regulation by transposases PTMs in lineages where the *Mos1* transposon is well established still remains an interesting question that is currently under investigation.

## SUPPLEMENTARY DATA

Supplementary Data are available at NAR Online, including [3,23].

## FUNDING

Francois Rabelais University (Tours - France); the Centre National de la Recherche Scientifique (CNRS); the French Ministère de l’Education Nationale, de la Recherche et de la Technologie; the Association Francaise contre les Myopathies [AFM # 11468 to CAG]; the Agence Nationale de la Recherche [ANR project Elegineer, # ANR 2010 BLAN 1618 02 to CAG]; Région Centre/FEDER grant [# 2699-33931, SyMBioMS] to the CBM-ICOA Federation (in part toward high-resolution spectrometry). Funding for open access charge: ANR project (Elegineer).

*Conflict of interest statement*. None declared.

## Supplementary Material

Supplementary Data
