# Supplementary material for: cAMP protein kinase phosphorylates the Mos1 transposase and regulates its activity: evidences from mass spectrometry and biochemical analyses
Source: Nucleic Acids Res. 2013 Sep 28;42(2):1117–28. doi: 10.1093/nar/gkt874 (PMC3902898; doi:10.1093/nar/gkt874)
Supplement: Supplementary Data [file supp_42_2_1117__index.html]

cAMP protein kinase phosphorylates the Mos1 transposase and regulates its activity: evidences from mass spectrometry and biochemical analyses — cAMP protein kinase phosphorylates the Mos1 transposase and regulates its activity: evidences from mass spectrometry and biochemical analyses — Supplementary Data 

# cAMP protein kinase phosphorylates the Mos1 transposase and regulates its activity: evidences from mass spectrometry and biochemical analyses

## Supplementary Data

files

**Files in this Data Supplement:**

- Supplementary Data - pdf file
