# Supplementary material for: cAMP protein kinase phosphorylates the Mos1 transposase and regulates its activity: evidences from mass spectrometry and biochemical analyses
Source: Nucleic Acids Res. 2013 Sep 28;42(2):1117–28. doi: 10.1093/nar/gkt874 (PMC3902898; doi:10.1093/nar/gkt874)
Supplement: Supplementary Data [file supp_gkt874_nar-01723-h-2013-File004.pdf]

Bouchet et al: Supplemental data

Supp Table 1. Oligonucleotides used in the main text

**Mos1 ITRs**
Uncleaved (UC-ITR)
5′CTAGAACTAGTGGATC**TATCAGGTGTACAAGTATGAAATGTCGTTT**GATCCCCCGGGCTGCAGC** 3′<br>3′ TTGATCACCTAG**ATAGTCCACATGTTCATACTTTACAGCAAA**CTAGGGGGCCCGACGTCGTTAA 5′
Pre-Cleaved (PC-ITR)
5′ **GGTGTACAAGTATGAAATGTCGTTT**GATCCCCCGGGCTGCAGC** 3′<br>3′ **AGTCCACATGTTCATACTTTACAGCAAA**CTAGGGGGCCCGACGTCGTTAA 5′
**Site directed mutagenesis**
S170A
**Up** **5′**CCT AAA CGT AAA AAG **GCA** TAC GTT GAT CCT **3′**<br>**Dw** **3′**GGA TTT GCA TTT TTC **CGT** ATG CAA CTA GGT **5′**
S170D
**Up** **5′**CCT AAA CGT AAA AAG **GAT** TAC GTT GAT CCT **3′**<br>**Dw** **3′**GGA TTT GCA TTT TTC **CTA** ATG CAA CTA GGT **5′**
S2D
**Up** **5′** GAG GGA AGG GTA ATG **GAC** AGT TTC GTG CCG **3′**<br>**Dw** **3′** CTC CCT TCC CAT TAC **CTG** TCA AAG CAC GGC **5′**

Mos1 ITRs: Oligonucleotides used for EMSAs. The sequence of the *Mos1* 3' ITR is indicated in bold. The TA dinucleotide is underlined. Stars located the labelling position.

Site directed mutagenesis: Oligonucleotides used for mutagenesis. Mutated bases are in bold. Mutagenesis was performed using the QuickChange Site-Directed Mutagenesis kit (Agilent Technologies).

Supplementary Figure 1. S170D is unable to support PEC assembly

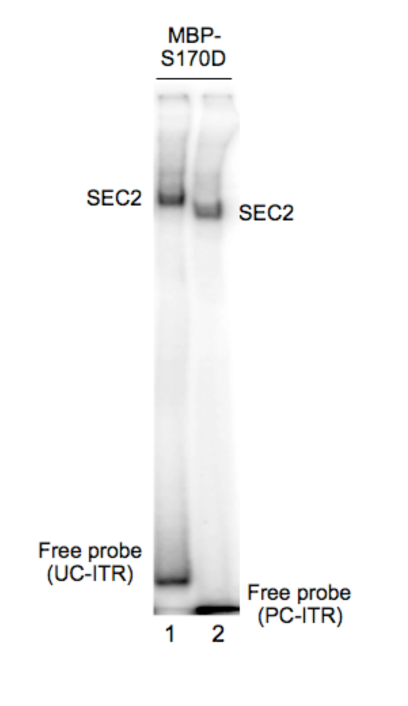

EMSAs were performed as described in the main text, using MBP-S170D with the *Mos1* 3'ITR as a probe, under conditions allowing either single-end complex (SEC2) assembly (lane 1) or paired-ends complex (PEC) assembly (lane 2). As previously shown, un-cleaved ITR (UC-ITR) only support SEC2 assembly. In contrast, pre-cleaved ITR (PC-ITR) supports either SEC2 or PEC assembly. The position of the complex observed when using the PC-ITR, compared to that of the complex observed when using the UC-ITR, indicates that this complex is indeed a SEC2 (Jaillet et al, PlosOne 2012, ref 3 of the main text). The complexes formed between S170D and the ITRs are identified in the margins.

Supplementary Figure 2. PKA treatment does not affect the cleavage activity of MBP-S170A

Time course analyses of excision were performed using MBP-S170A treated (right panel) or not (left panel) by PKA, and the pBC-3T3 plasmid as the donor of transposon. The assays were performed at 30°C, and the resulting products were loaded onto agarose gel. The various products are depicted on the right, and their positions on the gel are indicated. First strand nicking at one transposon end generated an open circular product (OC). Second strand nicking linearized the donor (L), yielding the single-end break product. A similar sequence of nicks at the other transposon end yielded the double-end break products, which consist of the plasmid backbone (B) plus the excised transposon fragment. SC: super coiled donor.

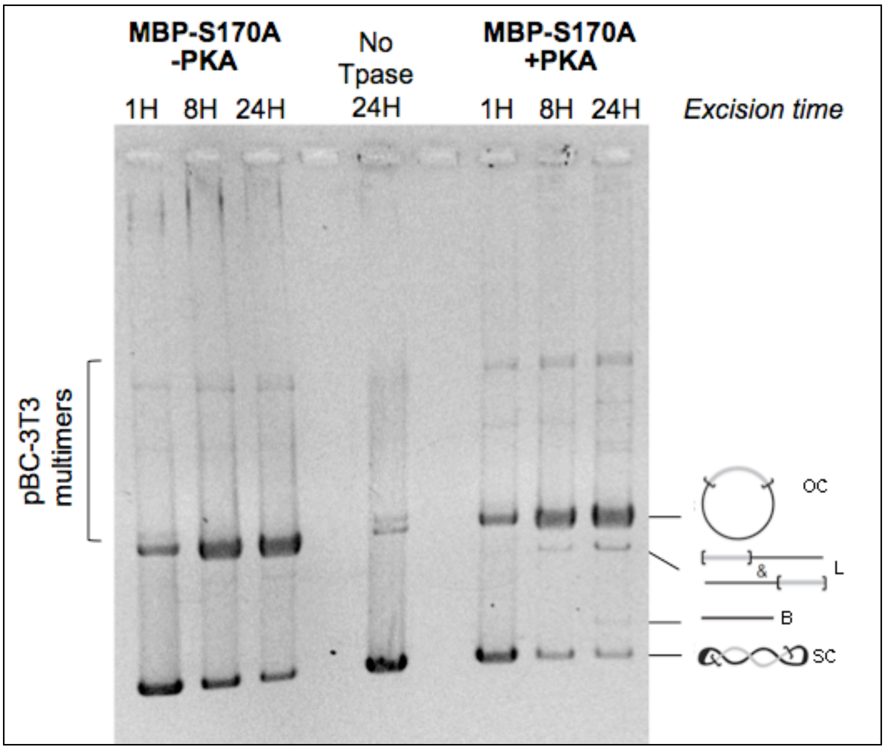

After treatment by PKA, MBP-S170A (unable to be phosphorylated at position 170 relative to the S170A mutation) was still able to promote *Mos1* excision (as demonstrated by the detection of the backbone product). However, the excision efficiency was dramatically reduced, compared to that of the wild type protein without phosphorylation, in agreement with their respective transposition rates: 10^{-5} for the wild type protein (after incubation and purification) and 10^{-6} for the mutant (see main text). This is also in agreement with the ability of MBP-S170A to promote PEC assembly (Fig. 4C, main text). The MBP-S170A –PKA seemed to achieve only non-specific cleavages, whereas it was still able to promote PEC assembly (Fig. 4C, main text).

Supplementary Figure 3. Sequence alignments of *mariner* Transposases mentioned in the main text

Sequences are shown from the WVPHEL motif (a landmark for mariner transposases)(in blue) to the residue 190. The first **D** of the catalytic triad is in red. The PKA consensus site for Threonine or Serine phosphorylation is underlined. The phosphorylable **residue** (at position 170 according to MOS1 numbering) is in green highlighted in yellow. Numbering is given according to the MOS1 sequence. The accession numbers are indicated in bold together with the host-species.

Mauritiana sub-family
(1): **X78906** (*D. mauritiana*) MOS1
(2): **AF035569** (*D. sechellia*)
(3): **X78907** (*D. simulans*)
(4): **AJ781771** (*M. bouvieri*)

Irritans sub-family
(5): **U11646** (*H. irritans*) HIMAR1
(6): **U11656** (*D. ananassae*)
(7): **U49974** (*H. sapiens*)

Cecropia sub-family
(8): HSMAR-Ra (*H. sapiens*) – (ancestral / Miskey et al, MCB 2007)(ref 23 of the main text)
(9): **U80776** (*H. sapiens*) SETMAR
(10): **M63844** (*H. cecropia*)
(11): **X79719** (*D. tigrina*)

mellifera/capitata sub-family
(12): **U19902** (*A. melifera*)
(13): **U40493** (*C. capitata*)
(14): **U08094** (*D. erecta*)

```
      119        130         140        150        160        170          180        190
(1)   WVPHELNERQMERRKNTCE--LILSRYKRKSFLHRIVTGDEKWIFFVSPKRKKS---YVDPGQPATSTARPNRFGKK
(2)   WVPHELNERQMERRKNTCE--ILLSRYKRKSFLHRIVTGDEKWIFFVNPKRKKS---YVDPGQPVTSTALPNRFGKK
(3)   WVPHELNERQMERRKNTCE--ILLSRYKRKSFLHRIVTGDEKWIFFVNPKRKKS---YVDPGQPATSTARPNRFGKK
(4)   WVPHELNDRQMENRKIVSE--MLLQRYERKSFLHRIVTGDGKWIYFENPKRKKS---WLSPGEAGPSTARPNRFGRK

(5)   WVPRELTFDQKQQRVDDSERCLLQLTRNTPEFFRRYVTMDETWLHHYTPESNRQSAEWTATGEPSPKRGKTQKSAGK
(6)   WVPRELTFDQKQQRVDDSERCLLQLTRNTPEFFRRYVTMDETFLHHYNPESNRQSAEWTATGEPTPKRGRTQKSPGK
(7)   WVPHQLSEKNKVDRMSTAI---LNWDQDPEAFLRRIVTGDETWLYQYDPEDKAQSKQWLPRGGSGPVKAKADWSRAK

(8)   WVPHELSENQKNRRFEVSS--SLILRNNNEPFLDRIVTCDEKWILYDN--RRRS-AQWLDREEAPKHFPKPNLHQKK
(9)   WVPHELTENQKNRRFEVSS--SLILRNHNEPFLDRIVTCDEKWILYDN--RRRS-AQWLDQEEAPKHFPKPILHPKK
(10)  WVPHELSESNLQTRVDCCV--LTLNRHNNEGILNRIITCDEKWILYDN--RKRS-SQWLNPGDPAKSCPKRKLTQKK
(11)  WVPHELKDEHKQQRLDACL--LSLSRNKADPFLHRIVTCDEKWIMYDN--RKRS-SQWLDPDEPPKHCPKRKVHQKK

(12)  WVPHELKETHLTQRINICD--LLKKRNENDPFLKRLITGDEKWVVYNNIKRKRS---WGRPGEPAQTTSKAGIHQKK
(13)  WVPHQLSEKNKVDRMSTAI--LSLRRIKNEPFLDRLLTGDEKWILYNNVQRKRT---WKQAHEGAEPMSKGGLHPMM
(14)  WVPHDLTQKNLLDRINACD--LMLKRNELDPFLKRMVTGDEKWITYDNIKRKRS---WSKAGESSQTVAKPGLTARK
```
